# *catena*-Poly[[diaqua­bis­(formato-κ*O*)nickel(II)]-μ-2,4,6-tris­(4-pyrid­yl)-1,3,5-triazine-κ^2^ *N* ^2^:*N* ^4^]

**DOI:** 10.1107/S1600536811012281

**Published:** 2011-04-13

**Authors:** Miao Feng, Hui-Juan Tian, Huai-Feng Mi, Tong-Liang Hu

**Affiliations:** a Biochemical Section of Key Laboratory of Functional Polymer Materials, The Ministry of Education of China, Chemical School of Nankai University, 300071 Tianjin, People’s Republic of China; b Department of Chemistry, Nankai University, Tianjin 300071, People’s Republic of China

## Abstract

In the title compound, [Ni(CHO_2_)_2_(C_18_H_12_N_6_)(H_2_O)_2_]_*n*_, the Ni^II^ ion, lying on a crystallographic inversion center, has a distorted octa­hedral coordination comprising two water ligands, two O-atom donors from formate ligands and two N-atom donors from the 2,4,6-tris­(4-pyrid­yl)-1,3,5-triazine ligands. These ligands bridge the Ni^II^ complex units, forming zigzag chains along the *c* axis. Adjacent chains are linked by O—H⋯O hydrogen bonds, forming a three-dimensional supra­molecular network.

## Related literature

For the structures and properties of coordination compounds with 2,4,6-tris­(4-pyrid­yl)-1,3,5-triazine as a ligand, see: Abrahams *et al.* (1999 ▶); Barrios *et al.* (2007 ▶); Batten *et al.* (1995 ▶); Dybtsev *et al.* (2004 ▶). 2~2~O ligand should bind through the O atom
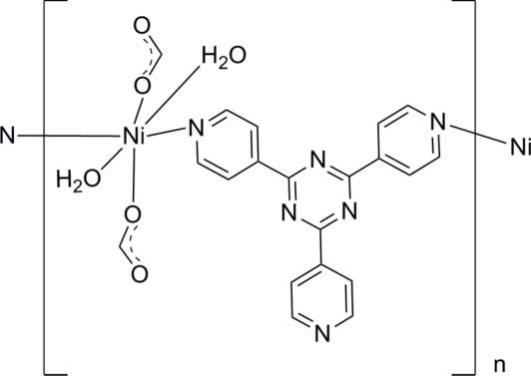


## Experimental

### 

#### Crystal data

[Ni(CHO_2_)_2_(C_18_H_12_N_6_)(H_2_O)_2_]
*M* *_r_* = 497.11
Monoclinic, 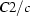

*a* = 24.725 (5) Å
*b* = 10.969 (2) Å
*c* = 7.4196 (15) Å
β = 90.23 (3)°
*V* = 2012.2 (7) Å^3^
*Z* = 4
Mo *K*α radiation
μ = 1.02 mm^−1^
*T* = 293 K
0.15 × 0.10 × 0.10 mm

#### Data collection

Rigaku SCX-mini diffractometer
Absorption correction: multi-scan (*ABSCOR*; Higashi, 1995 ▶) *T*_min_ = 0.836, *T*_max_ = 1.000
10365 measured reflections
2302 independent reflections
1937 reflections with *I* > 2σ(*I*)
*R*_int_ = 0.040

#### Refinement

*R*[*F* ^2^ > 2σ(*F* ^2^)] = 0.034
*wR*(*F* ^2^) = 0.078
*S* = 1.05
2302 reflections
153 parameters
H-atom parameters constrained
Δρ_max_ = 0.33 e Å^−3^
Δρ_min_ = −0.28 e Å^−3^

### 

Data collection: *PROCESS-AUTO* (Rigaku, 1998 ▶); cell refinement: *PROCESS-AUTO*; data reduction: *CrystalStructure* (Rigaku/MSC, 2002 ▶); program(s) used to solve structure: *SHELXS97* (Sheldrick, 2008 ▶); program(s) used to refine structure: *SHELXL97* (Sheldrick, 2008 ▶); molecular graphics: *SHELXTL* (Sheldrick, 2008 ▶); software used to prepare material for publication: *publCIF* (Westrip, 2010 ▶).

## Supplementary Material

Crystal structure: contains datablocks I, global. DOI: 10.1107/S1600536811012281/bq2289sup1.cif

Structure factors: contains datablocks I. DOI: 10.1107/S1600536811012281/bq2289Isup2.hkl

Additional supplementary materials:  crystallographic information; 3D view; checkCIF report

## Figures and Tables

**Table 1:** Hydrogen-bond geometry (Å, °)

*D*—H⋯*A*	*D*—H	H⋯*A*	*D*⋯*A*	*D*—H⋯*A*
O3—H6⋯O2^i^	0.86	1.96	2.818 (2)	177
O3—H7⋯O2^ii^	0.83	1.95	2.777 (2)	174

Symmetry codes: (i) 
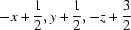
; (ii) 
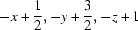
.

**Fig. 1.**
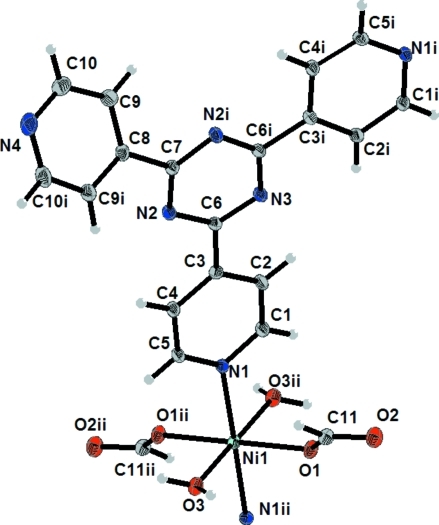
A fragment of the title compound showing the atom numbering scheme. Displacement ellipsoids are drawn at the 30% probability level. Symmetry codes: (i) (-x, -y, -z + 1.5); (ii) (-x + 1/2, -y + 1.5, -z + 2).

**Fig. 2.**
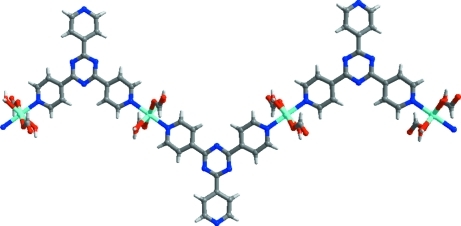
A view of the title structure along the c axis, showing the zigzag chain.

**Fig. 3.**
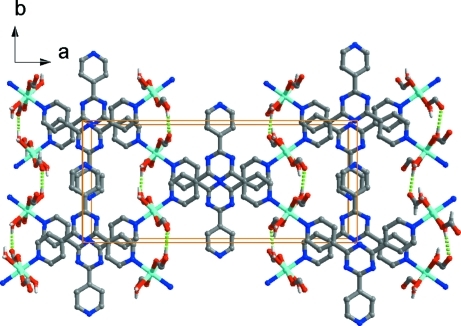
The crystal packing of the title compound. O—H···O hydrogen bonds are shown as dashed lines.

## supplementary crystallographic information

### Comment

As an interesting polydentate nitrogen donor ligand, 2,4,6-tris(4-pyridyl)-1,3,5-triazine has attracted increasing attention in the synthesis of novel transition metal complexes with novel topology and properties (Abrahams *et al.*, 1999; Dybtsev *et al.*, 2004; Barrios *et al.*, 2007; Batten *et al.*, 1995). Our interest in 2,4,6-tris(4-pyridyl)-1,3,5-triazine transition metal complexes prompts us to report the title compound (I).

As shown in Fig. 1, in the title compound, [Ni(C_18_H_12_N_6_)(H_2_O)_2_(HCOO)_2_]n, the Ni^II^ ion, lying on a crystallographic inversion center, has a distorted octahedral coordination sphere comprising two water ligands, two O-atom donors from formate ligands and two N-atom donors from the 2,4,6-tris(4-pyridyl)-1,3,5-triazine ligands. These ligands bridge the Ni^II^ complex units to form zigzag chains along *c* axis (Fig. 2). Adjacent chains are linked by O—H···O hydrogen bonds (Table 1), forming a three-dimensional supramolecular network (Fig. 3).

### Experimental

A mixture of Ni(HCOO)_2_.2H_2_O (0.15 mmol), 2,4,6-tris(4-pyridyl)-1,3,5-triazine (0.05 mmol), and 10 ml H_2_O were put in a 23-ml Teflon liner reactor and heated at 413 K in oven for 72 h. The resulting solution was slowly cooled to room temperature to yield single crystals of the title compound.

### Refinement

All H atoms were positioned geometrically (C—H = 0.93 Å) and allowed to ride on their parent atoms, with *U*_iso_(H) = 1.2*U*_eq_(parent atom). The H atoms of the water molecules were located in Fourier difference maps and refined isotropically.

### Crystal data

**Table:**

[Ni(CHO_2_)_2_(C_18_H_12_N_6_)(H_2_O)_2_]	*F*(000) = 1024
*M**_r_* = 497.11	*D*_x_ = 1.641 Mg m^−^^3^
Monoclinic, *C*2/*c*	Mo *K*α radiation, λ = 0.71073 Å
Hall symbol: -C 2yc	Cell parameters from 9569 reflections
*a* = 24.725 (5) Å	θ = 3.1–27.5°
*b* = 10.969 (2) Å	µ = 1.02 mm^−^^1^
*c* = 7.4196 (15) Å	*T* = 293 K
β = 90.23 (3)°	Block, green
*V* = 2012.2 (7) Å^3^	0.15 × 0.10 × 0.10 mm
*Z* = 4	

### Data collection

**Table:**

Rigaku SCX-mini diffractometer	2302 independent reflections
Radiation source: fine-focus sealed tube	1937 reflections with *I* > 2σ(*I*)
graphite	*R*_int_ = 0.040
ω scans	θ_max_ = 27.5°, θ_min_ = 3.1°
Absorption correction: multi-scan (*ABSCOR*; Higashi, 1995)	*h* = −31→32
*T*_min_ = 0.836, *T*_max_ = 1.000	*k* = −14→14
10365 measured reflections	*l* = −9→9

### Refinement

**Table:**

Refinement on *F*^2^	Primary atom site location: structure-invariant direct methods
Least-squares matrix: full	Secondary atom site location: difference Fourier map
*R*[*F*^2^ > 2σ(*F*^2^)] = 0.034	Hydrogen site location: inferred from neighbouring sites
*wR*(*F*^2^) = 0.078	H-atom parameters constrained
*S* = 1.05	*w* = 1/[σ^2^(*F*_o_^2^) + (0.0334*P*)^2^ + 2.3183*P*] where *P* = (*F*_o_^2^ + 2*F*_c_^2^)/3
2302 reflections	(Δ/σ)_max_ = 0.001
153 parameters	Δρ_max_ = 0.33 e Å^−^^3^
0 restraints	Δρ_min_ = −0.28 e Å^−^^3^

### Special details

**Table:**

Geometry. All e.s.d.'s (except the e.s.d. in the dihedral angle between two l.s. planes) are estimated using the full covariance matrix. The cell e.s.d.'s are taken into account individually in the estimation of e.s.d.'s in distances, angles and torsion angles; correlations between e.s.d.'s in cell parameters are only used when they are defined by crystal symmetry. An approximate (isotropic) treatment of cell e.s.d.'s is used for estimating e.s.d.'s involving l.s. planes.
Refinement. Refinement of *F*^2^ against ALL reflections. The weighted *R*-factor *wR* and goodness of fit *S* are based on *F*^2^, conventional *R*-factors *R* are based on *F*, with *F* set to zero for negative *F*^2^. The threshold expression of *F*^2^ > σ(*F*^2^) is used only for calculating *R*-factors(gt) *etc*. and is not relevant to the choice of reflections for refinement. *R*-factors based on *F*^2^ are statistically about twice as large as those based on *F*, and *R*- factors based on ALL data will be even larger.

### Fractional atomic coordinates and isotropic or equivalent isotropic displacement parameters (Å^2^)

**Table:**

	*x*	*y*	*z*	*U*_iso_*/*U*_eq_	
Ni1	0.2500	0.7500	1.0000	0.01798 (11)	
O1	0.22888 (6)	0.64439 (12)	0.78618 (18)	0.0263 (3)	
O2	0.19168 (7)	0.61015 (14)	0.5190 (2)	0.0337 (4)	
O3	0.29896 (6)	0.86926 (12)	0.85264 (19)	0.0263 (3)	
H6	0.3027	0.9430	0.8884	0.039*	
H7	0.3001	0.8711	0.7403	0.039*	
N1	0.18044 (6)	0.86186 (14)	0.9633 (2)	0.0204 (4)	
N2	0.04510 (7)	1.19452 (15)	0.8071 (2)	0.0251 (4)	
N3	0.0000	1.0076 (2)	0.7500	0.0245 (5)	
N4	0.0000	1.6408 (2)	0.7500	0.0428 (7)	
C1	0.13264 (8)	0.81007 (18)	0.9265 (3)	0.0241 (4)	
H1	0.1299	0.7257	0.9355	0.029*	
C2	0.08733 (8)	0.87491 (18)	0.8760 (3)	0.0248 (4)	
H2	0.0551	0.8349	0.8495	0.030*	
C3	0.09060 (8)	1.00126 (18)	0.8654 (3)	0.0210 (4)	
C4	0.13930 (8)	1.05662 (18)	0.9086 (3)	0.0239 (4)	
H4	0.1427	1.1410	0.9053	0.029*	
C5	0.18282 (8)	0.98373 (18)	0.9569 (3)	0.0240 (4)	
H5	0.2154	1.0215	0.9864	0.029*	
C6	0.04255 (8)	1.07256 (18)	0.8045 (3)	0.0208 (4)	
C7	0.0000	1.2503 (3)	0.7500	0.0229 (6)	
C8	0.0000	1.3860 (3)	0.7500	0.0252 (6)	
C9	−0.04675 (9)	1.4505 (2)	0.7877 (3)	0.0327 (5)	
H9	−0.0791	1.4101	0.8097	0.039*	
C10	−0.04418 (10)	1.5768 (2)	0.7918 (4)	0.0400 (6)	
H10	−0.0750	1.6194	0.8257	0.048*	
C11	0.20837 (8)	0.67816 (19)	0.6412 (3)	0.0246 (4)	
H11	0.2052	0.7617	0.6226	0.029*	

### Atomic displacement parameters (Å^2^)

**Table:**

	*U*^11^	*U*^22^	*U*^33^	*U*^12^	*U*^13^	*U*^23^
Ni1	0.01931 (18)	0.01621 (18)	0.01840 (18)	0.00276 (15)	−0.00433 (12)	−0.00092 (15)
O1	0.0344 (8)	0.0217 (7)	0.0227 (7)	0.0047 (6)	−0.0086 (6)	−0.0032 (6)
O2	0.0428 (9)	0.0349 (9)	0.0233 (8)	0.0010 (7)	−0.0093 (7)	−0.0037 (7)
O3	0.0350 (8)	0.0216 (7)	0.0224 (7)	−0.0013 (6)	0.0005 (6)	0.0019 (6)
N1	0.0188 (8)	0.0193 (8)	0.0231 (8)	0.0024 (7)	−0.0044 (6)	0.0001 (7)
N2	0.0212 (9)	0.0180 (8)	0.0362 (10)	0.0011 (7)	−0.0062 (7)	−0.0005 (7)
N3	0.0193 (12)	0.0187 (12)	0.0353 (14)	0.000	−0.0059 (10)	0.000
N4	0.0506 (19)	0.0189 (14)	0.059 (2)	0.000	−0.0079 (15)	0.000
C1	0.0238 (10)	0.0154 (10)	0.0332 (11)	−0.0003 (8)	−0.0034 (8)	0.0014 (8)
C2	0.0191 (10)	0.0196 (10)	0.0357 (12)	−0.0029 (8)	−0.0057 (8)	0.0002 (9)
C3	0.0192 (9)	0.0210 (10)	0.0226 (10)	0.0023 (8)	−0.0023 (8)	−0.0013 (8)
C4	0.0228 (10)	0.0159 (9)	0.0330 (11)	0.0000 (8)	−0.0052 (8)	−0.0007 (8)
C5	0.0181 (10)	0.0222 (10)	0.0316 (11)	−0.0020 (8)	−0.0050 (8)	−0.0036 (9)
C6	0.0178 (9)	0.0196 (10)	0.0249 (10)	0.0007 (8)	−0.0012 (8)	−0.0009 (8)
C7	0.0224 (13)	0.0167 (13)	0.0297 (14)	0.000	−0.0021 (11)	0.000
C8	0.0285 (15)	0.0185 (14)	0.0284 (15)	0.000	−0.0066 (12)	0.000
C9	0.0278 (11)	0.0236 (11)	0.0466 (14)	0.0024 (9)	−0.0025 (10)	0.0023 (10)
C10	0.0407 (14)	0.0256 (12)	0.0536 (16)	0.0108 (11)	−0.0052 (12)	−0.0010 (11)
C11	0.0275 (11)	0.0234 (10)	0.0228 (10)	0.0023 (8)	−0.0015 (8)	0.0002 (8)

### Geometric parameters (Å, °)

**Table:**

Ni1—O1^i^	2.0309 (14)	C1—C2	1.378 (3)
Ni1—O1	2.0309 (14)	C1—H1	0.9300
Ni1—O3^i^	2.0934 (14)	C2—C3	1.391 (3)
Ni1—O3	2.0935 (14)	C2—H2	0.9300
Ni1—N1	2.1293 (16)	C3—C4	1.385 (3)
Ni1—N1^i^	2.1294 (16)	C3—C6	1.491 (3)
O1—C11	1.244 (2)	C4—C5	1.387 (3)
O2—C11	1.244 (2)	C4—H4	0.9300
O3—H6	0.8557	C5—H5	0.9300
O3—H7	0.8343	C7—N2^ii^	1.339 (2)
N1—C1	1.338 (3)	C7—C8	1.489 (4)
N1—C5	1.339 (3)	C8—C9^ii^	1.385 (3)
N2—C7	1.339 (2)	C8—C9	1.385 (3)
N2—C6	1.339 (3)	C9—C10	1.387 (3)
N3—C6^ii^	1.332 (2)	C9—H9	0.9300
N3—C6	1.332 (2)	C10—H10	0.9300
N4—C10	1.336 (3)	C11—H11	0.9300
N4—C10^ii^	1.336 (3)		
			
O1^i^—Ni1—O1	180.0	C1—C2—H2	120.6
O1^i^—Ni1—O3^i^	95.50 (6)	C3—C2—H2	120.6
O1—Ni1—O3^i^	84.50 (6)	C4—C3—C2	118.34 (18)
O1^i^—Ni1—O3	84.50 (6)	C4—C3—C6	122.08 (18)
O1—Ni1—O3	95.50 (6)	C2—C3—C6	119.57 (18)
O3^i^—Ni1—O3	180.0	C3—C4—C5	118.69 (18)
O1^i^—Ni1—N1	88.64 (6)	C3—C4—H4	120.7
O1—Ni1—N1	91.36 (6)	C5—C4—H4	120.7
O3^i^—Ni1—N1	87.62 (6)	N1—C5—C4	123.41 (18)
O3—Ni1—N1	92.38 (6)	N1—C5—H5	118.3
O1^i^—Ni1—N1^i^	91.36 (6)	C4—C5—H5	118.3
O1—Ni1—N1^i^	88.64 (6)	N3—C6—N2	125.14 (19)
O3^i^—Ni1—N1^i^	92.39 (6)	N3—C6—C3	116.04 (18)
O3—Ni1—N1^i^	87.61 (6)	N2—C6—C3	118.82 (17)
N1—Ni1—N1^i^	180.0	N2^ii^—C7—N2	125.7 (3)
C11—O1—Ni1	127.47 (13)	N2^ii^—C7—C8	117.17 (13)
Ni1—O3—H6	119.3	N2—C7—C8	117.17 (13)
Ni1—O3—H7	124.0	C9^ii^—C8—C9	118.5 (3)
H6—O3—H7	106.4	C9^ii^—C8—C7	120.74 (14)
C1—N1—C5	117.10 (16)	C9—C8—C7	120.74 (14)
C1—N1—Ni1	119.57 (13)	C8—C9—C10	118.5 (2)
C5—N1—Ni1	122.98 (13)	C8—C9—H9	120.8
C7—N2—C6	114.35 (18)	C10—C9—H9	120.8
C6^ii^—N3—C6	115.4 (2)	N4—C10—C9	123.8 (2)
C10—N4—C10^ii^	116.6 (3)	N4—C10—H10	118.1
N1—C1—C2	123.56 (18)	C9—C10—H10	118.1
N1—C1—H1	118.2	O2—C11—O1	125.8 (2)
C2—C1—H1	118.2	O2—C11—H11	117.1
C1—C2—C3	118.84 (18)	O1—C11—H11	117.1
			
O3^i^—Ni1—O1—C11	125.73 (18)	C3—C4—C5—N1	0.3 (3)
O3—Ni1—O1—C11	−54.27 (18)	C6^ii^—N3—C6—N2	−0.22 (15)
N1—Ni1—O1—C11	38.26 (18)	C6^ii^—N3—C6—C3	−179.8 (2)
N1^i^—Ni1—O1—C11	−141.74 (18)	C7—N2—C6—N3	0.4 (3)
O1^i^—Ni1—N1—C1	−137.13 (16)	C7—N2—C6—C3	180.00 (15)
O1—Ni1—N1—C1	42.87 (16)	C4—C3—C6—N3	173.55 (17)
O3^i^—Ni1—N1—C1	−41.57 (15)	C2—C3—C6—N3	−5.0 (3)
O3—Ni1—N1—C1	138.43 (15)	C4—C3—C6—N2	−6.1 (3)
O1^i^—Ni1—N1—C5	49.91 (16)	C2—C3—C6—N2	175.33 (19)
O1—Ni1—N1—C5	−130.09 (16)	C6—N2—C7—N2^ii^	−0.19 (13)
O3^i^—Ni1—N1—C5	145.47 (16)	C6—N2—C7—C8	179.82 (13)
O3—Ni1—N1—C5	−34.53 (16)	N2^ii^—C7—C8—C9^ii^	−145.57 (15)
C5—N1—C1—C2	2.8 (3)	N2—C7—C8—C9^ii^	34.43 (15)
Ni1—N1—C1—C2	−170.57 (17)	N2^ii^—C7—C8—C9	34.43 (15)
N1—C1—C2—C3	−1.2 (3)	N2—C7—C8—C9	−145.57 (15)
C1—C2—C3—C4	−0.9 (3)	C9^ii^—C8—C9—C10	−2.10 (17)
C1—C2—C3—C6	177.77 (19)	C7—C8—C9—C10	177.90 (17)
C2—C3—C4—C5	1.3 (3)	C10^ii^—N4—C10—C9	−2.33 (18)
C6—C3—C4—C5	−177.31 (19)	C8—C9—C10—N4	4.5 (4)
C1—N1—C5—C4	−2.3 (3)	Ni1—O1—C11—O2	−173.47 (16)
Ni1—N1—C5—C4	170.79 (16)		

Symmetry codes: (i) −*x*+1/2, −*y*+3/2, −*z*+2; (ii) −*x*, *y*, −*z*+3/2.

### Hydrogen-bond geometry (Å, °)

**Table:**

*D*—H···*A*	*D*—H	H···*A*	*D*···*A*	*D*—H···*A*
O3—H6···O2^iii^	0.86	1.96	2.818 (2)	177
O3—H7···O2^iv^	0.83	1.95	2.777 (2)	174

Symmetry codes: (iii) −*x*+1/2, *y*+1/2, −*z*+3/2; (iv) −*x*+1/2, −*y*+3/2, −*z*+1.
